# Remote ischaemic preconditioning: closer to the mechanism?

**DOI:** 10.12688/f1000research.9633.1

**Published:** 2016-12-13

**Authors:** Jonathan M. Gleadle, Annette Mazzone

**Affiliations:** 1 School of Medicine, Flinders University, Adelaide, Australia; 2 Department of Renal Medicine, Flinders Medical Centre, Adelaide, Australia; 3 Cardiac Surgery Research and Perfusion, Cardiac and Thoracic Surgical Unit, Flinders Medical Centre, Adelaide, Australia

**Keywords:** ischaemia, reperfusion, remote ischaemic preconditioning, cardioprotection

## Abstract

Brief periods of ischaemia followed by reperfusion of one tissue such as skeletal muscle can confer subsequent protection against ischaemia-induced injury in other organs such as the heart. Substantial evidence of this effect has been accrued in experimental animal models. However, the translation of this phenomenon to its use as a therapy in ischaemic disease has been largely disappointing without clear evidence of benefit in humans. Recently, innovative experimental observations have suggested that remote ischaemic preconditioning (RIPC) may be largely mediated through hypoxic inhibition of the oxygen-sensing enzyme PHD2, leading to enhanced levels of alpha-ketoglutarate and subsequent increases in circulating kynurenic acid (KYNA). These observations provide vital insights into the likely mechanisms of RIPC and a route to manipulating this mechanism towards therapeutic benefit by direct alteration of KYNA, alpha-ketoglutarate levels, PHD inhibition, or pharmacological targeting of the incompletely understood cardioprotective mechanism activated by KYNA.

## Introduction

Ischaemia followed by reperfusion of one tissue such as muscle can confer subsequent protection against ischaemia-induced injury in other organs such as the heart. Substantial evidence of this effect has been accrued in experimental animal models, but the translation to a therapy in ischaemic disease has not been definitively achieved in humans. Furthermore, experimental evidence for a large number of potential mediators and mechanisms has been obtained, but a clear understanding of the mechanisms is lacking. This commentary focuses on recent work examining a novel mechanism that may underlie remote ischaemic preconditioning (RIPC) ^1^.

## What is ischaemic preconditioning?

Ischaemic preconditioning is the phenomenon whereby brief periods of ischaemia followed by tissue reperfusion confer subsequent protection against ischaemia-induced injury. The concept, proposed 30 years ago by Murry *et al*., demonstrated that brief cycles of ischaemia and reperfusion of the coronary arteries protect the myocardium from subsequent prolonged ischaemia and reperfusion, leading to a reduction in infarct size ^2^.

## What is remote ischaemic preconditioning?

The concept was developed further with the observation that ischaemia in one coronary territory could protect cardiac tissue supplied by other epicardial arteries ^3^. Birnbaum *et al*. went on to demonstrate that “remote” transient ischaemia of non-myocardial tissues could also be associated with reductions in the extent of myocardial infarction. They combined partial reduction of blood flow to the hindlimb with increased oxygen demand by rapid electrical stimulation of the gastrocnemius muscle and showed reduced myocardial infarct size in rabbits ^4^. Subsequently, Kharbanda *et al.* showed similar beneficial effects in a porcine model of myocardial infarction and applied the concept of RIPC to healthy human volunteers by inducing transient non-invasive ischaemia with the use of a blood pressure cuff applied to one arm and demonstrated improved endothelial function in the contralateral arm ^5^.

## Does it benefit patients?

The important clinical question has emerged of whether RIPC can be used therapeutically in the wide range of medical conditions in which ischaemic injury occurs. RIPC has been applied in elective cardiac surgery, vascular surgery, percutaneous coronary intervention, and organ transplantation in attempts to improve cardiac, renal, and other outcomes. Individual, small randomised controlled trials have been reported to show potential benefit ^6– 9^. Hu *et al*. undertook a systematic review of 30 randomised controlled trials to investigate the effects of RIPC on the incidence and outcomes of acute kidney injury (AKI) and found evidence of benefit in preventing contrast-induced AKI ^10^. However, there was not benefit in ischaemia reperfusion-induced AKI ^10^, and more recent trials have also failed to see clear benefit in that setting ^11^. The REmote preconditioning for Protection Against Ischaemia-Reperfusion in renal transplantation (REPAIR) trial found some evidence that RIPC using transient arm ischaemia-reperfusion improved renal transplant function ^12^.

In the setting of cardiac surgery, meta-analyses have not confirmed any therapeutic benefit from RIPC ^13^ nor have more recent larger-scale studies. The Effect of RIPC on Clinical Outcomes in Coronary Artery Bypass Graft (CABG) Surgery (ERICCA) study, a randomised controlled clinical trial in 1,612 patients, showed no effect of RIPC on clinical outcomes ^14^. RIPC consisting of four 5-minute cycles of ischaemia-reperfusion of the upper arm did not improve clinical outcomes in patients undergoing elective CABG. No differences were seen in mortality, stroke, myocardial infarction, or AKI. The RIPC for Heart Surgery (RIPHeart) trial of 1,385 patients used a similar upper limb ischaemia protocol but also failed to see benefit ^15^.

Overall, these results are disappointing but convincing in their failure to see a therapeutic benefit of RIPC in most patients. The optimum type, duration, and timing of the ischaemic intervention is uncertain; skeletal muscle mass, hepatic function, concurrent medications, choice of anaesthetic, and the effect on different target organs may also vary and influence the effect of the intervention. How can the benefits seen in experimental studies be translated to a useful therapy, and does RIPC operate in humans? Understanding the mechanism of effect might enable optimisation of the clinical use of RIPC.

## What is the mechanism of remote ischaemic preconditioning?

Whilst definitive evidence for therapeutic benefit in humans is lacking, evidence that experimental manipulations can have a protective benefit is strong (for review, see 16). A large number of different mechanisms have been suggested, including roles for neurally mediated mechanisms and hormonal mediators (for selected examples, see Table 1), with a recent workshop suggesting that the mechanisms underlying RIPC remain unclear ^17^. Recent work has implicated the hypoxia response and the generation of circulating molecular mediators. Hypoxia is a central component of ischaemia, and the hypoxia-inducible factor (HIF) transcription factors play a dominant role in co-ordinating the transcriptional response to hypoxia. The abundance of the HIF-α factors is controlled by oxygen-dependent prolyl hydroxylation by the PHD family of 2-oxoglutarate dioxygenases ^18– 20^ (PHD1, 2, and 3, also known as EGLN2, 1, and 3, respectively) and their transcriptional potency by the FIH-1 asparaginyl hydroxylase ^21, 22^. Several studies have implicated the HIF–PHD system in the mechanism of RIPC. These include impaired RIPC in mice heterozygous for a knockout allele encoding HIF-1α ^23^, activation of HIF-1α by ischaemic preconditioning, and enhancement of cardiac protection by pharmacological and genetic enhancement of HIF-1α ^24^. Mice with genetically reduced levels of PHD2 (and hence enhanced HIF-1α levels) showed greater resistance to cardiac ischaemia ^25, 26^, as did animals with activation of HIF by pharmacological PHD inhibition or VHL deficiency ^27^, though other studies have suggested that HIF-1α upregulation is unnecessary in acute RIPC ^28^.

**Table 1.** Selected animal studies that have implicated potential mechanisms and mediators of remote ischaemic preconditioning of the heart (RIPC).

Potential Mechanism/Mediator	Species	RIPC model	Reference
Neurally mediated erythropoietin release	Mice	Hindlimb ischaemia	29
MicroRNA-144	Mice	Hindlimb ischaemia	36
Neurally mediated bradykinin release	Rat	Mesenteric artery occlusion	37
Adenosine	Rat	Mesenteric artery occlusion	38
Bradykinin and epoxyeicosatrienoic acids	Dog	Abdominal skin incision	39
Endogenous opioids	Rat	Mesenteric artery occlusion	40
SDF-1/CXCR4	Rat	Hindlimb ischaemia	31
Adenosine and ATP- sensitive potassium (KATP) channels	Rabbit	Renal ischaemia	41
Haem oxygenase-1	Rat	Hindlimb ischaemia	42
Interleukin-10	Mice	Hindlimb ischaemia	23
Nitrite	Mice	Hindlimb ischaemia	43
Apolipoprotein A-I	Rat	Hindlimb ischaemia	44
Glucagon-like peptide-1	Rat	Hindlimb ischaemia	45
Hypoxia inducible factor (HIF)	Mice	Hindlimb ischaemia	23

There are a broad array of HIF-mediated responses to hypoxia that might help mediate ischaemic preconditioning, including the promotion of anaerobic metabolism, vascularity, and vasodilatation, reactive oxygen species protection, and alterations in cell survival and cell cycle. Some of these HIF-dependent hypoxic responses include the release of circulating mediators by ischaemic tissue, such as its canonical target erythropoietin ^29, 30^ and others including CXCL12 (SDF-1) ^31^, that might act as circulating mediators of RIPC. Whilst these studies do suggest a role for the HIF–PHD system in RIPC, they have not fully disentangled the requirement for HIF activation in the remote ischaemic tissue versus that in the target protected organ nor the relative contributions of neural or hormonal mediators.

## Is there a role for kynurenic acid as the mediator of remote ischaemic preconditioning?

A major insight into the mechanism of RIPC and the role of the HIF–PHD system and circulating mediators has come from the recent work of Kaelin and colleagues ^1^. They initially provided further evidence for the protective effects of HIF activation by showing that genetic and chronic PHD2 inactivation in mice hearts conferred protective benefit against permanent and transient cardiac ischemia. Similar beneficial effects were also seen with acute systemic PHD2 genetic inactivation and with systemic administration of a pharmacological PHD inhibitor. To determine whether manipulations of the HIF–PHD system in the remote ischaemic tissue (but not the target heart) affected RIPC, they studied mice with PHD2 inactivated only in skeletal muscle. Such mice again showed enhanced myocardial protection following ischaemia. They then undertook parabiotic experiments to provide important evidence that this protective effect was mediated by a circulating factor. To determine the nature of this circulating factor, they tested for cytokine and metabolite differences in the blood of mice with and without PHD2 skeletal muscle inactivation. No significant changes were seen in cytokines or molecules such as erythropoietin, which has previously been suggested to act as a circulating mediator of RIPC. Similarly, no plausible secreted candidates were identified from genetic expression analyses between mice with and without PHD2 skeletal muscle inactivation. However, when blood was compared by analysis with liquid chromatography and mass spectroscopy, significant differences in tryptophan metabolites were observed. Similar alterations were also seen in blood shortly after pharmacological PHD inhibition with significant elevations in the level of the tryptophan metabolite kynurenic acid (KYNA).

**Figure 1.**
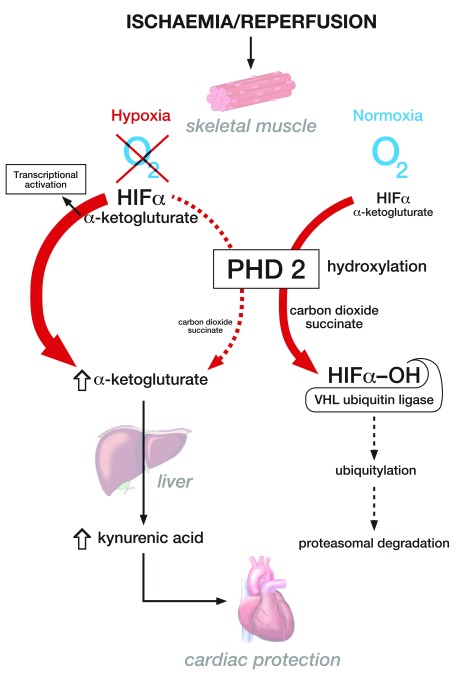
Schematic illustration of the pathways involved in enhanced kynurenic acid (KYNA) generation by the hypoxia-inducible factor (HIF) hydroxylase PHD2 during hypoxia. The figure demonstrates the mechanism by which muscle hypoxia results in the inhibition of PHD2 function leading to enhanced alpha-ketoglutarate generation and kynurenic acid production, which may mediate a cardioprotective effect. It also shows the canonical role of PHD2 in normoxia in the oxygen-dependent degradation of the transcription factor HIF. HIFα, hypoxia inducible factor α; PHD2, prolyl hydroxylase domain 2; VHL, von Hippel Lindau.

Further evidence implicating KYNA as a mediator of ischaemic preconditioning were obtained by abrogating RIPC with inhibitors of the tryptophan pathway and from the beneficial effects of administration of KYNA itself. Studies were then undertaken to explore the mechanism by which PHD2 inactivation in muscle resulted in increases in circulating KYNA and mediation via an increase in levels of the obligatory PHD co-substrate alpha-ketoglutarate with subsequent hepatic generation of KYNA ( Figure 1). Systemic alpha-ketoglutarate administration also protected hearts from ischaemia-reperfusion injury. PHD2 inhibition appeared to increase alpha-ketoglutarate levels as a consequence of its reduced decarboxylation, with evidence provided of a high rate of PHD2-dependent alpha-ketoglutarate conversion to succinate. This is superficially surprising given its well-understood role as an oxygen-sensing enzyme as opposed to one with significant roles in metabolic flux. Some support for a role for the kynurenine pathway in the mechanism of RIPC has been provided by studies in humans and rats in which circulating metabolites, including kynurenine and glycine, that demonstrated elevated levels after RIPC were injected prior to myocardial infarction and had a protective effect ^32^.

## Conclusions

These findings provide vital insights into a potential mechanism of RIPC and generate intriguing questions ( Box 1). Notably, what is the mechanism of the cardioprotective effect and does it operate in other tissues? Is it mediated through metabolic effects, via effects on specific G-protein-coupled receptors ^33^, or by the known influence of KYNA on the aryl hydrocarbon receptor (AHR) response ^34^ (which shares with the HIF pathway the heterodimeric transcription factor AHR nuclear translocator [ARNT])? Whilst protective effects of AHR activation have been suggested in some models of ischaemia, in others activation of the AHR response by tryptophan metabolites can have deleterious effects ^35^.

Box 1. **Outstanding questions concerning remote ischaemia preconditioning (RIPC) and the role of kynurenic acid (KYNA)**

- What is the mechanism of the cardioprotective effect, and does it operate in other tissues?
- Can the manipulation of KYNA or alpha-ketoglutarate levels or direct pharmacological targeting of the cardioprotective mechanism activated by KYNA produce therapeutic benefit in patients with ischaemic diseases?
- What mass of tissue ischaemia is necessary to achieve sufficient perturbations in the levels of circulating KYNA?
- Will the emerging PHD inhibitors currently being trialled for their erythropoietic effect ^46^ have protective benefits?
- Do the other known influences on PHD function, such as oxygen availability, iron and ascorbate, or perturbations of alpha-ketoglutarate metabolism, influence protective mechanisms via this effect *in vivo*?
- What effect is produced by acute versus chronic elevations in the levels of such molecules, and to what extent do metabolic compensations or the complex feedback loops operating in the PHD–hypoxia-inducible factor (HIF) system affect the operation of RIPC?
- Does ischaemia operate locally to mediate protective effects through this mechanism?
- Does this mechanism operate in other situations, such as hypoxic tumours?
- Are there associations between levels of KYNA and outcomes in ischaemic diseases?
- Is KYNA the dominant mediator of RIPC in humans, or are other mediators/mechanisms more important?

What is the relative importance of this newly defined mechanism of RIPC to other pathways, how is it related to neurally mediated effects, and how do they interact? Can manipulation of KYNA or alpha-ketoglutarate levels or direct pharmacological targeting of the cardioprotective mechanism activated by KYNA produce therapeutic benefit in patients with ischaemic diseases? In contrast to the impressive protective benefits seen in the work of Olenchock and colleagues ^1^ and other animal studies, does the failure of RIPC to achieve improved clinical outcomes reflect inadequate suppression of PHD2 activity and/or insufficient increases in levels of KYNA? Improved understanding of the transduction of the RIPC signal from remote tissue to protected target may now allow improvements in clinical strategies to deliver the enormous potential benefits of RIPC and the development of new pharmacological approaches that directly activate the protective pathway.
